# Maternal Betaine Supplementation Mitigates Maternal High Fat Diet-Induced NAFLD in Offspring Mice through Gut Microbiota

**DOI:** 10.3390/nu15020284

**Published:** 2023-01-06

**Authors:** Liuqiao Sun, Xuying Tan, Xiaoping Liang, Hangjun Chen, Qian Ou, Qiongmei Wu, Xinxue Yu, Hanqing Zhao, Qiaoli Huang, Zehua Yi, Jun Wei, Feng Wu, Huilian Zhu, Lijun Wang

**Affiliations:** 1 Department of Maternal, Child and Adolescent Health, School of Medicine, Jinan University, Guangzhou 510632, China; 2 Department of Child Health Care, Guangzhou Women and Children’s Medical Center, Guangzhou Medical University, Guangdong Provincial Clinical Research Center for Child Health, Guangzhou 510623, China; 3 Department of Nutrition, School of Medicine, Jinan University, Guangzhou 510632, China; 4 Department of Science and Technology, Guangzhou Customs, Guangzhou 510623, China; 5 Department of Nutrition, School of Public Health, Sun Yat-Sen University, Guangzhou 510080, China

**Keywords:** non-alcoholic fatty liver disease, betaine, gut–liver axis, gut microbiota, SCFAs

## Abstract

Maternal betaine supplementation has been proven to alleviate non-alcoholic fatty liver disease (NAFLD) in offspring caused by maternal high-fat diet (MHFD). The gut–liver axis plays an important role in NAFLD pathogenesis. However, whether maternal betaine supplementation can alleviate NAFLD in offspring by the gut–liver axis is unknown. C57BL/6J mice were fed with high-fat diet for 4 weeks before mating, and supplemented with 1% betaine during pregnancy and lactation. After weaning, offspring mice were fed with standard diet to 10 weeks. Maternal betaine supplementation reduced hepatic triglyceride content and alleviated hepatic steatosis in offspring mice exposed to MHFD. Furthermore, the mRNA expression of *PPARα*, *CPT1α* and *FATP2* was increased and *TNFα* was reduced by maternal betaine supplementation. Maternal betaine intake decreased the relative abundances of *Proteobateria*, *Desulfovibrio* and *Ruminococcus*, but increased the relative abundances of *Bacteroides* and *Parabacteroides*. Moreover, maternal betaine intake increased the concentrations of short-chain fatty acids (SCFAs), including acetic acid, butyric acid and valeric acid, in the feces. Gut microbiota and SCFAs were significantly correlated with hepatic triglyceride content and expression of the above genes. Maternal betaine intake had no effect on other gut microbiota-related metabolites (bile acid and trimethylamine-n-oxide). Altogether, maternal betaine supplementation ameliorated MHFD-induced NAFLD possibly through regulating gut microbiota and SCFAs in offspring mice.

## 1. Introduction

Non-alcoholic fatty liver disease (NAFLD) represents the most common cause of chronic liver disease and remains a major public health problem, which is estimated to affect 25% of the world population and 7.6% of children [1,2]. Epidemiological studies have linked maternal overnutrition and obesity in utero with fetal development and the risk profile for disease later in life [3,4]. The risk of biopsy-proven NAFLD in offspring born to mothers with overweight and obesity in early pregnancy was increased by 51% and 226%, respectively [5]. An animal study also found that exposure to maternal high-fat diet (MHFD) led to NAFLD in adulthood among offspring mice [6]. The adverse effects of MHFD on liver last over three generations [7]. However, the underlying mechanisms and effective interventions remain uncertain.

The gut microbiota consists of a large community of microorganisms residing in the gastrointestinal tract, which influence the development and function of a metabolic system. A large body of literature supports the notion that there is a bidirectional communication system between the gut and the liver, known as the gut–liver axis [8,9]. NAFLD severity was related to gut microbiota dysbiosis and disorder of gut flora-related metabolites [10]. Our previous study found that the serum level of trimethylamine-n-oxide (TMAO), which is the metabolite of choline through conversion of the gut microbiota and hepatic flavin monooxygenases, was positively correlated with the grade of liver steatosis in NAFLD patients [11]. The excessive accumulation of bile acid also has a toxic effect on liver cells. The content of total bile acid (TBA) in NAFLD patients was significantly higher than that in non-NAFLD patients [12]. Moreover, short-chain fatty acids (SCFAs) engaged with the modulation of NAFLD. Studies have observed that acetate, propionate and butyrate supplementation can inhibit hepatic lipid accumulation and exert anti-inflammatory properties [13,14,15]. Given the emerging reports on the influences of maternal obese status on the offspring’ microbiome, it seems possible that MHFD-induced changes in the microbiome may be related to metabolic disorder in offspring [16]. Indeed, the experiment of fecal microbiota transplantation evidenced that maternal obesity can impact the gut microbiota in offspring, increasing susceptibility to NAFLD [17]. Therefore, improving maternal nutrition status and then remodeling the offspring gut microbiota is a promising strategy to break the cross generational transmission of metabolic diseases.

Betaine, also named N-trimethylglycine, is naturally abundant in beet, whole grains and some vegetables. Our previous studies have reported that betaine plays a protective role in NAFLD mice induced by HFD and ApoE knockout [18,19]. Dietary betaine improved lipid accumulation in the white adipose tissues, liver and muscle through elevating Akkermansia muciniphila (AKK) and increased the contents of acetate and butyrate in the feces [20]. In addition, the effects of maternal betaine intake during pregnancy on progeny health have received increasing attention. Towards healthy outcomes, a cohort study based in Singapore found that a higher maternal plasma betaine concentration was associated with smaller infant birth weight and less abdominal fat [21]. The metabolic regulation of maternal betaine intake on offspring is also related to the abundance of AKK. More betaine intake during lactation led to higher betaine content in the breastmilk, thus an increased the abundance of AKK in offspring’ feces, leading to lower adiposity [22]. Maternal betaine supplementation alleviated glucose metabolism disorders in 4- and 20-week-old offspring mice induced by MHFD, accompanied by persistent alteration of the gut microbiota [23]. However, whether the protective effects of maternal betaine intake on NAFLD in offspring are related to gut microbiota regulation and its related metabolites is still unknown.

In this study, we aimed to investigate the beneficial effect of maternal betaine supplementation on NAFLD in offspring mice induced by MHFD during pregnancy and lactation. Moreover, we explored the alterations of the gut microbiota and its related metabolites. The findings of the present study will facilitate a better understanding of the transgenerational effect of MHFD on offspring from the view of the gut microbiota, and further provide a theoretical basis for utilization of betaine in the pregnant and parturient population to reduce the risk of NAFLD in their offspring.

## 2. Materials and Methods

### 2.1. Animals Experiment

Both C57BL/6J female and male mice at 6 weeks old were purchased from the Zhejiang Vital River Laboratory Animal Technology Co., Ltd. (licensed ID: SCXK 2019-0001, Jiaxing, China). The standard diet (H10010) and high-fat diet (H10060) were purchased from Beijing Huafukang Biotechnology Co., Ltd. (Beijing, China), and betaine (B802317) was purchased from Shanghai Macklin Biochemical Technology Co., Ltd (Shanghai, China). The standard diet consisted of 10% kcal fat, 70% kcal carbohydrates, and 20% kcal protein. The high-fat diet consisted of 60% kcal fat, 20% kcal carbohydrates, and 20% kcal protein. All mice were housed in the experimental animal center of Jinan University under a 12 h light/dark cycle at 20–25 °C. After acclimatization for 1 week on a standard diet, the mice were randomly divided into the following groups: a control diet (CON) group, a high-fat diet (HFD) group, and a HFD + 1% betaine (HFD-BET) group. The mice in the CON group were fed with standard diet. The mice in other groups were fed with HFD. After a 4-week intervention, female mice were mated with male mice (1 male: 2 female). Then, the mice in the HFD-BET group were fed with water containing 1% wt/vol betaine throughout pregnancy and lactation. All pregnant mice were fed the indicated diet until offspring mice reached 3 weeks age. After weaning, the offspring mice were then given a standard diet to 10 weeks of age. During the experiment, the body weights of mice were monitored weekly. Mice were sacrificed after fasting for 12 h, and their blood, liver, intestine and its contents were collected. Some of the liver and colon tissues were saved in 4% paraformaldehyde for histopathology analysis and others were stored at −80 °C until analysis.

### 2.2. Glucose and Insulin Tolerance Tests (GTT/ITT)

The GTT and the ITT were preformed on gestation day 15 or 16, respectively. Female mice were fasted for 6 h and intraperitoneally injected 2 g/kg body weight glucose or 0.75 U/kg body weight insulin. Then, the blood glucose level was analyzed at 0, 15, 30, 60, 90, 120 min by a glucometer (590, Yuwell, Danyang, China). The areas under curves (AUC) were calculated by the trapezoidal integration.

### 2.3. Serum and Liver Biochemical Analysis

The serum levels of alanine aminotransferase (ALT), aspartate aminotransferase (AST), triglyceride (TG), total cholesterol (TC), high-density lipoprotein cholesterol (HDL-C), low-density lipoprotein cholesterol (LDL-C) and glucose (GLU) were measured by an automated chemical analyzer. The hepatic levels of TC and TG were measured using commercial assay kits (E1025 for TG, E1026 for TC, APPLYGEN, Beijing, China). The TBA level in the serum and liver were quantified by a commercial kit (E003-2-1, Nanjing Jiancheng Bioengineering Institute, Nanjing, China).

### 2.4. Histopathology Examination

The tissues of liver and colon were fixed with 4% paraformaldehyde at room temperature for 24 h. Then, they were embedded in paraffin, and sliced for regular hematoxylin-eosin (H&E) to analyze the change in pathology of tissues under confocal microscopy. Liver samples were also stained with oil red O to observe lipid accumulation.

### 2.5. Quantification of One-Carbon Metabolites in the Serum

The serum levels of betaine, choline and TMAO were quantified using high-performance liquid chromatography, as described in our previous work [19].

### 2.6. Quantification of SCFAs Content in the Cecal

SCFAs assays of fecal samples were performed by a gas chromatography–mass spectrometry system (GC–MS). Specifically, 50 mg samples were mixed with 50 μL of 15% phosphoric acid, 100 μL of 125 μg/mL isohexanoic acid solution (internal standard) and 400 μL ether. We centrifuged the homogenate at 4 °C 12,000 rpm for 10 min and extracted the supernatant for GC–MS analysis. An Agilent HP-INNOWAX capillary column (30 m length, 0.25 mm internal diameter and 0.25 µm of film thickness) was utilized for the separation. The temperature program of chromatography were as follows: the initial temperature was 90 °C, it was increased to 120 °C at 10 °C/min, and then raised to 150 °C at 5 °C/min. Finally, the temperature was increased to 250 °C at a rate of 25 °C/min for 2 min. The helium carrier gas flow rate was set at 1 mL/min. The energy of electron impact ionization in the mass spectrum was 70 eV.

### 2.7. 16S rRNA Pyrosequencing Analysis

Fecal bacterial DNA was isolated using cetyltrimethylammonium bromide (CTAB) method. The distinct regions (V3–V4) of the bacterial 16S rRNA gene was amplified using the 341F (5′-CCTAYGGGRBGCASCAG-3′) and 806R (5′-GGACTACNNGGGTATCTAAT-3′) primers. The PCR protocol was 98 °C for 1 min, followed by 30 cycles of 98 °C for 10 s, 50 °C for 30 s, and 72 °C for 30 s; and then remained at 72 °C for 10 min. Sequencing libraries were conducted by a TruSeq ^®^ DNA PCR-Free Sample Preparation Kit (Illumina, San Diego, CA, USA) and the sequence was detected using NovaSeq 6000 PE250 (Illumina, San Diego, CA, USA). The resulting sequences were grouped into operational taxonomic units (OTUs) with 97% similarity using QIIME2 software for further bioinformatics analysis. Kruskal–Wallis tests were used to identify the relative abundances of the gut microbiota at the phylum and genus levels. The Chao1, Shannon and Simpson indices were used to evaluate the alpha diversity, and the unweighted UniFrac index was used to evaluate the beta diversity, which was then displayed by non-metric dimensional scaling (NMDS). The differential taxa of the gut microbiota among groups were then identified by linear discriminant analysis effect size (LEfSe).

### 2.8. RNA Extraction and Quantitative Real-Time Polymerase Chain Reaction (qRT-PCR)

Total RNA of the tissue was extracted using a Trizol RNA Extraction Kit (9108, TaKaRa, Osaka, Japan), and then transcribed to first-strand complementary DNA (cDNA) using the reverse transcription reagent kit (RR036A, TaKaRa, Osaka, Japan). qRT-PCR was carried out on a CFX96 Real-Time PCR Detection System with a SYBR Premix Ex Taq kit (RR820A, TaKaRa, Osaka, Japan). β-actin was used as an internal control to normalize the gene expression. The primer sequences used in this study are summarized in Table 1.

### 2.9. Statistical Analysis

Data are presented as the means ± standard errors of the means (means ± SEM), and statistical significance was performed by one-way analysis of variance (ANOVA) using SPSS 24.0 statistical software. When the variance homogeneity test was satisfied, the Bonferroni *t*-test was applied; Otherwise, the Dunnett *t*-test was applied. The relationships between the levels of the gut microbiota and SCFAs and NAFLD-related parameters were determined by Spearman’s correlation coefficient test. *p* < 0.05 was considered statistically significant. GraphPad Prism 7.0 was used to generate graphs.

## 3. Results

### 3.1. Body Weight, Organ Coefficients and Serum Biochemistry in Dams and Offspring Mice

**Figure 1:**
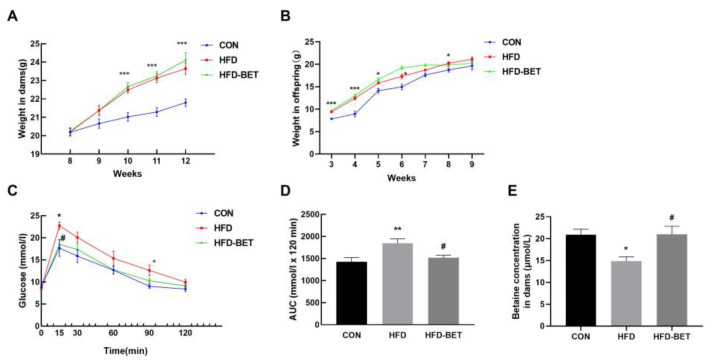
Body weight in dams and offspring mice and the GTT in dams. (**A**) Body weight in dams; (**B**) body weight in offspring mice; (**C**) serum glucose levels for the GTT in dams; (**D**) the AUC for the GTT in dams; (**E**) serum betaine levels in dams. Data are presented as the means ± SEM. * *p* < 0.05, ** *p* < 0.01 and *** *p* < 0.001 for the HFD group vs. the CON group; ^#^ *p* < 0.051 for the HFD-BET group vs. the HFD group. Abbreviation: CON group, control diet group; HFD group, high-fat diet group; HFD-BET group, high-fat diet + 1% betaine group.

As expected, MHFD significantly increased the body weight of dams and offspring mice compared with the CON group (Figure 1A,B). The viscera weight was measured after the mice were sacrificed. In dams, the visceral fat weight (*p* = 0.047) and visceral fat/body weight ratio (*p* = 0.042) were significantly increased in the HFD group compared with the CON group. In comparison with the HFD group, the liver weight (*p* = 0.008) and liver/body weight ratio (*p* = 0.036) in the HFD-BET group were significantly decreased (Table S1). In offspring mice, the liver weight (*p* = 0.017), liver/body weight ratio (*p* = 0.030), the visceral fat weight (*p* < 0.001) and visceral fat/body weight ratio (*p* < 0.001) were significantly increased in the HFD group compared with the CON group, and maternal betaine intake significantly reversed these indicators (Table 2). There was no difference in the water intake during pregnancy and lactation (Figure S1A,B). The betaine consumption of dams during pregnancy and lactation is shown in Figure S1C. The serum betaine level in the HFD group was lower than that in the CON group (14.86 ± 0.97 vs. 20.91 ± 1.24, *p* = 0.049), which was significantly inversed by betaine supplementation in dams (21.02 ± 1.80 vs. 14.86 ± 0.97, *p* = 0.039) (Figure 1E). The litter size was not significantly different among groups (Figure S2). Then, we detected the serum biochemistry in dams and offspring. In dams, the serum levels of TC (*p* = 0.033) and HDL-C (*p* = 0.018) in the HFD group were significantly higher than in the CON group; however, betaine supplementation had no effect on serum biochemistry (Table S1). In offspring mice, compared with the CON group, the serum levels of ALT (*p* = 0.018), AST (*p* = 0.014), TC (*p* = 0.002) and TG (*p* = 0.024) in the HFD group were significantly increased. Maternal betaine supplementation significantly reduced serum AST level (*p* = 0.014) (Table 2). These data demonstrated that MHFD induced body weight gain, liver weight gain, viscera weight gain, hyperlipidemia and liver injury in dams and offspring mice, while maternal betaine supplementation reversed the liver and viscera weight gain and liver injury in offspring mice.

We used the GTT and the ITT on gestation days 15 and 16, respectively. The glucose levels at 15 min (22.74 ± 0.85 vs. 17.68 ± 1.85, *p* = 0.030) and 90 min (12.62 ± 1.19 vs. 9.00 ± 0.49, *p* = 0.017) in the HFD group were significantly higher than in the CON group, and this was the same for the AUC values (1845.83 ± 98.55 vs. 1427.42 ± 92.30, *p* = 0.005). Betaine supplementation significantly reduced glucose levels at 15 min (17.98 ± 1.00 vs. 22.74 ± 0.85, *p* = 0.028) and the AUC values (1516.38 ± 58.75 vs. 1845.83 ± 98.55, *p* = 0.020) (Figure 1C,D). There was no difference in glucose levels and AUC in the ITT (Figure S3). These results suggest that betaine supplementation has a protective effect against MHFD-induced hyperglycemia in dams.

### 3.2. Maternal Betaine Intake Attenuated NAFLD in Offspring Mice Induced by MHFD

H&E staining confirmed prominent fatty liver in the HFD group compared with the CON group. The steatosis in the HFD group was more serious than that in the CON group. Oil red O results indicate that the HFD group had a denser lipid droplet than the CON group. However, these changes were alleviated by maternal betaine supplementation. Consistent with histological results, the hepatic levels of TG (403.42 ± 46.42 vs. 225.74 ± 31.16, *p* = 0.041) and TC (42.65 ± 3.56 vs. 28.25 ± 2.48, *p* = 0.014) in the HFD group were higher than that in the CON group, which were inversed by maternal betaine supplementation (206.97 ± 15.45 vs. 403.42 ± 46.42, *p* = 0.028 for TG; 22.12 ± 2.66 vs. 42.65 ± 3.56, *p* < 0.001 for TC).

**Figure 2:**
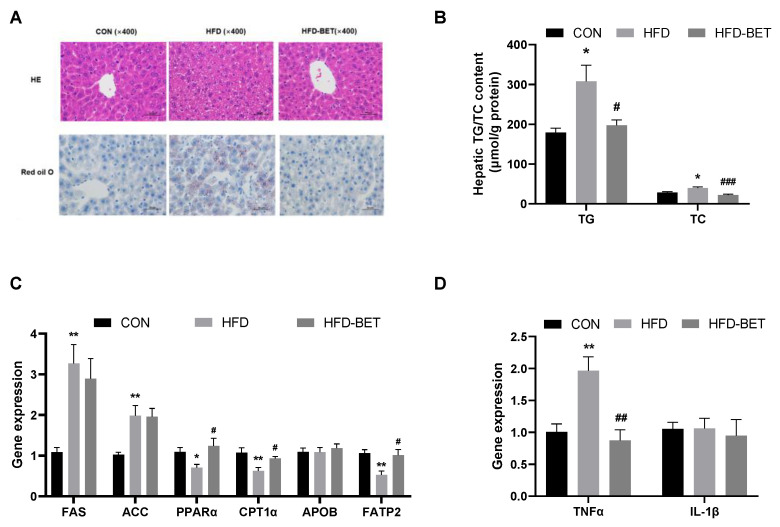
Maternal betaine supplementation attenuated NAFLD in offspring mice under MHFD. (**A**) Hematoxylin–eosin (H&E)- and oil red O-stained liver sections; (**B**) hepatic concentrations of TG and TC; (**C**) the mRNA expression of genes related to hepatic lipid metabolism; (**D**) the mRNA expression of genes related to hepatic inflammation. Data are presented as the means ± SEM. * *p* < 0.05 and ** *p* < 0.01 for the HFD group vs. the CON group; ^#^ *p* < 0.05, ^##^ *p* < 0.01 and ^###^ *p* < 0.001 for the HFD-BET group vs. the HFD group. Abbreviation: CON group, control diet group; HFD group, high-fat diet group; HFD-BET group, high-fat diet + 1% betaine group; TG, triglyceride; TC, total cholesterol.

The mRNA levels of genes associated with lipid metabolism were detected. For hepatic lipogenic genes, the mRNA levels of *FAS* (3.27 ± 0.46 vs. 1.05 ± 0.09, *p* = 0.003) and *ACC* (1.98 ± 0.25 vs. 1.03 ± 0.06, *p* = 0.006) were both increased in the HFD group compared with the CON group (Figure 2). For lipid oxidation-related genes, the mRNA expressions of *PPARα* (0.71 ± 0.08 vs. 1.09 ± 0.11, *p* = 0.022) and *CPT1α* (0.63 ± 0.08 vs. 1.10 ± 0.11, *p* = 0.009) were significantly decreased in the HFD group compared with the CON group, and maternal betaine supplementation increased the mRNA expression of *PPARα* (1.24 ± 0.18 vs. 0.71 ± 0.08, *p* = 0.047) and *CPT1α* (0.93 ± 0.05 vs. 0.63 ± 0.08, *p* = 0.027). Moreover, for lipid transport-related genes, *FATP2* mRNA expression was significantly decreased in the HFD group (0.53 ± 0.09 vs. 1.06 ± 0.08, *p* = 0.001) compared with the CON group, while betaine supplementation significantly increased the mRNA expression of *FATP2* (1.01 ± 0.14 vs. 0.53 ± 0.09, *p* = 0.022). As for inflammation-related genes, the mRNA expression of *TNFα* was elevated in the HFD group (1.97 ± 0.22 vs. 1.01 ± 0.12, *p* = 0.001) compared with the CON group, which was remarkably decreased by administration of betaine (0.87 ± 0.17 vs. 1.97 ± 0.22, *p* = 0.001). The mRNA expression of *APOB48* and *IL-1β* was not altered among groups. These data indicate that MHFD induced NAFLD in offspring mice through dysregulation of lipid metabolism and inflammation, which were significantly attenuated by maternal betaine supplementation.

### 3.3. Maternal Betaine Intake Altered the Gut Microbiota and Its Related Metabolites in Offspring Mice

#### 3.3.1. Maternal Betaine Intake Reversed the Disruption of Intestinal Development and Gut Barrier in Offspring Mice Exposed to MHFD

**Figure 3:**
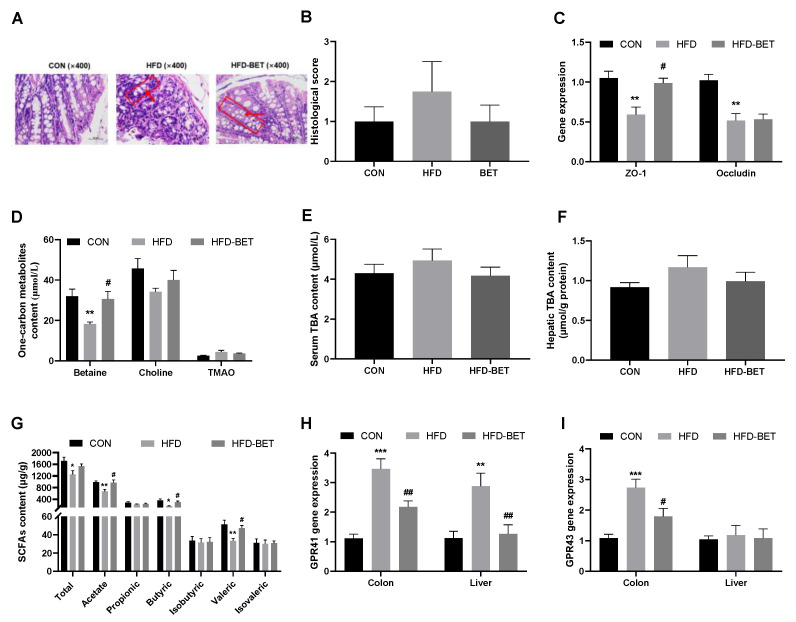
Effects of maternal betaine supplementation on intestinal barrier integrity and levels of gut flora-related metabolites. (**A**) Hematoxylin–eosin (H&E)-stained colon sections; the red box refers to a crypt, and the red arrow refers to a goblet cell; (**B**) histopathological score of colon; (**C**) the mRNA expression of *ZO-1* and *Occludin* in the colon; (**D**) one-carbon metabolites content in the serum; (**E**) TBA content in the serum; (**F**) TBA content in the liver; (**G**) SCFAs content in the cecal; (**H**,**I**) the mRNA expression of *GPR41/43* in the colon and liver. Data are presented as the means ± SEM. * *p* < 0.05, ** *p* < 0.01 and *** *p* < 0.001 for the HFD group vs. the CON group; ^#^ *p* < 0.05 and ^##^ *p* < 0.01 for the HFD-BET group vs. the HFD group. Abbreviation: CON group, control diet group; HFD group, high-fat diet group; HFD-BET group, high-fat diet + 1% betaine group; TMAO, trimethylamine-n-oxide; TBA, total bile acid; SCFAs, short-chain fatty acids.

The H&E staining of colon tissue indicates that the number of goblet cells and crypts was reduced and the crypt structure was destroyed in the HFD group, and maternal betaine supplementation improved the colon structure (Figure 3A). The histopathology score of colon tissue showed a downward trend after maternal betaine intake (Figure 3B). Compared with the CON group, the mRNA expression of *ZO-1* (0.59 ± 0.10 vs. 1.05 ± 0.09, *p* = 0.002) and *Occludin* (0.52 ± 0.09 vs. 1.02 ± 0.07, *p* = 0.001) was significantly decreased in the HFD group, while maternal betaine supplementation increased the mRNA expression of *ZO-1* (0.99 ± 0.06 vs. 0.59 ± 0.10, *p* = 0.009) (Figure 3C). These data indicate that maternal betaine supplementation reversed the disruption of intestinal development and integrity of gut barrier in offspring mice exposed to MHFD.

#### 3.3.2. Maternal Betaine Supplementation Reversed Gut Microbiota-Related Metabolites in Offspring Mice Exposed to MHFD

The levels of total SCFAs (1257.93 ± 122.49 vs. 1717.20 ± 128.74, *p* = 0.027), acetic acid (665.83 ± 72.43 vs. 991.92 ± 42.07, *p* = 0.005), butyric acid (162.70 ± 14.28 vs. 360.03 ± 53.37, *p* = 0.026) and valeric acid (28.44 ± 5.34 vs. 51.33 ± 4.70, *p* = 0.008) in the HFD group were significantly lower than those in the CON group, while maternal betaine supplementation significantly increased the contents of acetic acid (937.09 ± 77.01 vs. 665.83 ± 72.43, *p* = 0.028), butyric acid (300.45 ± 36.88 vs. 162.70 ± 14.28, *p* = 0.044) and valeric acid (47.12 ± 2.99 vs. 28.44 ± 5.34, *p* = 0.031) (Figure 3G). GPR41 and GPR43 were highly expressed in the colon tissue. Compared with the CON group, the mRNA expression of *GPR41* (3.51 ± 0.34 vs. 1.13 ± 0.14, *p* < 0.001) and *GPR43* (2.73 ± 0.28 vs. 1.08 ± 0.13, *p* < 0.001) in the colon of the HFD group were markedly increased, which were reversed by maternal betaine supplementation (Figure 3H,I). The mRNA expression of *GPR41* in the liver among groups showed a similar trend with colon (Figure 3H). Serum betaine concentration was significantly reduced in the HFD group compared with the CON group (18.29 ± 0.93 vs. 31.97 ± 3.58, *p* = 0.005), and was attenuated by maternal betaine supplementation (30.60 ± 3.71 vs. 18.29 ± 0.93, *p* = 0.044). In addition, maternal betaine supplementation has no significant effect on the serum levels of choline and TMAO (Figure 3D). Maternal betaine supplementation did not affect the TBA level in the liver and serum of offspring mice (Figure 3E,F). These above data indicate that maternal betaine supplementation increased SCFAs levels in the feces of offspring mice, while the contents of TMAO and TBA were not changed.

#### 3.3.3. Maternal Betaine Supplementation Altered the Gut Microbiota in Offspring Mice Exposed to MHFD

**Figure 4:**
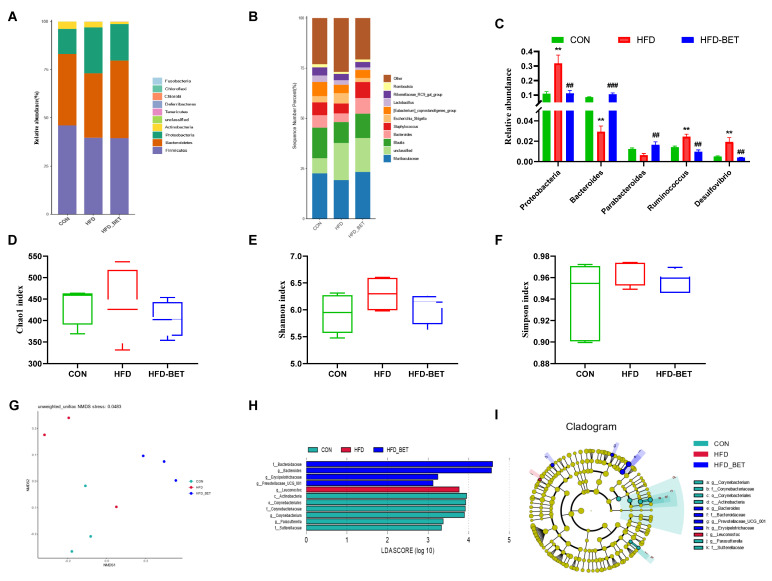
Effects of maternal betaine intake on gut microbiota in offspring mice. (**A**) Taxonomic composition distribution at the phylum level; (**B**,**C**) taxonomic composition distribution at the genus level; (**D**–**F**) alpha diversity analysis; (**G**) non-metric dimensional scaling (NMDS) analysis; (**H**,**I**) linear discriminant analysis effect size (LEfSe) analysis, and the linear discriminant analysis (LDA) score using a threshold score larger than 2.0. Data are presented as the means ± SEM. ** *p* < 0.01 for the HFD group vs. the CON group; ^##^ *p* < 0.01 and ^###^ *p* < 0.001 for the HFD-BET group vs. the HFD group. Abbreviation: CON group, control diet group; HFD group, high-fat diet group; HFD-BET group, high-fat diet + 1% betaine group.

In offspring mice, the dominant gut microbiota at the phylum level among the three groups are shown in Figure 4A, which showed that the HFD-BET group tended to have a higher abundance of *Bacteroidetes*. Figure 4B shows the relative abundance of top 10 genera. At the phylum level, the relative abundance of the *Proteobacteria* phylum was significantly increased in the HFD group (0.32 ± 0.06 vs. 0.11 ± 0.01, *p* = 0.002), and maternal betaine intake reversed this change (0.11 ± 0.02 vs. 0.32 ± 0.06, *p* = 0.004) (Figure 4C). At the genus level, a significant increase in *Ruminococcus* (0.024 ± 0.002 vs. 0.014 ± 0.001, *p* = 0.003) and *Desulfovibrio* (0.019 ± 0.004 vs. 0.005 ± 0.001, *p* = 0.007) was also observed in the HFD group, while maternal betaine supplementation reduced the relative abundance of *Ruminococcus* (0.010 ± 0.002 vs. 0.024 ± 0.002, *p* = 0.001) and *Desulfovibrio* (0.004 ± 0.000 vs. 0.019 ± 0.004, *p* = 0.005). In addition, the relative abundance of *Bacteroides* (0.106 ± 0.009 vs. 0.029 ± 0.006, *p *< 0.001) and *Parabacteroides* (0.016 ± 0.003 vs. 0.006 ± 0.002, *p* = 0.008) was significantly increased after maternal betaine supplementation (Figure 4C). The alpha diversity was estimated the Chao1, Shannon and Simpson indices, and there was no significant difference among the three groups (Figure 4D,F). The results of beta diversity presented that three clusters were relatively separated, which suggests that the gut microbiota composition was different among the three groups (Figure 4E). Additionally, LEfSe analysis was further used to compare the microbial composition among groups. As shown in Figure 4H,I, in the CON group, the *Parasutterella* genus within *Sutterellaceae*, and the *Corynebacterium* genus, within the *Corynebacteriaceae* family, within the *Corynebacteriales* order within the *Actinobacteria* class were enriched. The HFD group had a higher relative abundance of *Leuconostoc* at the genus level. In addition, the HFD-BET group mainly increased the relative abundance of the *Bacteroides* genus within the *Bacteroidaceae* family, and *Prevotellaceae_UCG_001*, the *Erysipelotrichaceae* genus. These changes in the gut microbiota in offspring mice were different from those in dams. The effects of betaine intake on the gut microbiota in dams are shown in Figure S4. These results demonstrate that maternal betaine supplementation reshaped the gut microbiota in offspring mice exposed to MHFD.

#### 3.3.4. Association of the Altered Gut Microbiota and SCFAs with NAFLD-Related Parameters in Offspring Mice

**Figure 5:**
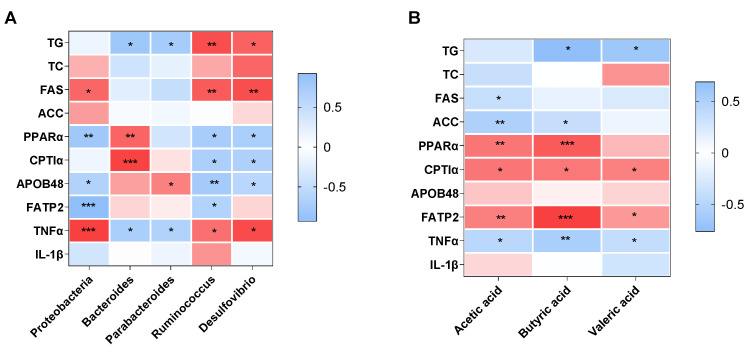
Heatmap of the correlation analysis of the altered gut microbiota (**A**) and SCFAs levels (**B**) with the NAFLD-related parameters in offspring mice. * *p* < 0.05, ** *p* < 0.01 and *** *p* < 0.001 represent a significant correlation. Abbreviation: TG, triglyceride; TC, total cholesterol.

Figure 5A shows the relationship between the gut microbiota and NAFLD-related parameters. The hepatic TG level was positively associated with the relative abundance of *Bacteroides* (r = −0.815, *p* = 0.014) and *Parabacteroides* (r = −0.755, *p* = 0.030), and negatively related to the relative abundance of *Ruminococcus* (r = 0.848, *p* = 0.002) and *Desulfovibrio* (r = 0.755, *p* = 0.030). The *Proteobacteria* phylum was positively associated with the mRNA expression of *FAS* (r = 0.734, *p* = 0.016) and *TNFα* (r = 0.920, *p* < 0.001), and negatively associated with the mRNA expression of *PPARα* (r = −0.802, *p* = 0.005), *APOB48* (r = −0.659, *p* = 0.010) and *FATP2* (r = −0.932, *p* < 0.001). The *Bacteroides* genus showed significantly positive relationships with the mRNA expression of *PPARα* (r = 0.745, *p* = 0.002) and *CPT1α* (r = 0.910, *p* < 0.001), and inverse relationships with the mRNA expression of *TNFα* (r = −0.706, *p* = 0.033). There were significant relationships between the abundance of *Parabacteroides* and the mRNA expression of *APOB48* (r = 0.606, *p* = 0.037) and *TNFα* (r = −0.669, *p* = 0.034). The abundance of *Ruminococcus*/*Desulfovibrio* was negatively correlated with the mRNA expression of *PPARα* (r = −0.732, *p* = 0.016 for *Ruminococcus*; r = −0.711, *p* = 0.021 for *Desulfovibrio*), *CPT1α* (r = −0.701, *p* = 0.035 for *Ruminococcus*; r = −0.688, *p* = 0.028 for *Desulfovibrio*) and *APOB48* (r = −0.764, *p* = 0.002 for *Ruminococcus*; r = −0.591, *p* = 0.026 for *Desulfovibrio*), and positively correlated with the mRNA expression of *FAS* (r = 0.793, *p* = 0.006 for *Ruminococcus*; r = 0.852, *p* = 0.002 for *Desulfovibrio*) and *TNFα* (r = 0.685, *p* = 0.029 for *Ruminococcus*; r = 0.869, *p* = 0.011 for *Desulfovibrio*). In addition, there were significant correlations between *Ruminococcus* and the mRNA expression of *FATP2* (r = −0.652, *p* = 0.022).

Figure 5B shows the association between SCFAs levels and NAFLD-related parameters. The hepatic TG level showed significantly inverse relationships with the concentration of butyric acid (r = −0.762, *p* = 0.029) and valeric acid (r = −0.671, *p* = 0.024). Acetic acid and butyric acid were positively correlated with the mRNA expression of *PPARα* (r = 0.495, *p* = 0.005 for acetic acid; r = 0.589, *p* = 0.001 for butyric acid), *CPT1α* (r = 0.492, *p* = 0.011 for acetic acid; r = 0.584, *p* = 0.036 for butyric acid) and *FATP2* (r = 0.462, *p* = 0.005 for acetic acid; r = 0.670, *p* < 0.001 for butyric acid), and negatively correlated with the mRNA expression of *ACC* (r = 0.584, *p* = 0.002 for acetic acid; r = 0.584, *p* = 0.002 for butyric acid) and *TNFα* (r = −0.480, *p* = 0.015 for acetic acid; r = −0.580, *p* = 0.009 for butyric acid). In addition, acetic acid was positively correlated with the mRNA expression of *FAS* (r = −0.392, *p* = 0.032). The valeric acid level was positively associated with the mRNA expression of *CPT1α* (r = 0.458, *p* = 0.028) and *FATP*2 (r = 0.368, *p* = 0.035), and inversely associated with *TNF*α mRNA expression (r = −0.406, *p* = 0.049).

## 4. Discussion

Studies have shown that gut microbiota disorder can lead to metabolic diseases, including obesity, type 2 diabetes mellitus and NAFLD [24,25,26]. Maternal overweight and obesity are related to an altered gut microbiome of their offspring at birth, 1 month, 6 months and 2 years old [27,28,29]. In addition, a previous animal model found that the disturbance of the infant gut microbiota caused by MHFD persisted into adulthood [30]. The change in the gut microbiota in offspring has been evidenced to further affect the occurrence and development of NAFLD through the gut–liver axis [31]. Previous studies have shown that maternal betaine supplementation, starting in early life, provides long-term protection from NAFLD in offspring induced by HFD and glucocorticoid in dams, and the underlying mechanisms may be attributed to the regulation of lipogenic genes [32,33]. However, due to the complex, multidirectional pathophysiology involved in NAFLD, how maternal betaine supplementation affects genes involved in lipid metabolism and NAFLD in offspring remains unknown. In the present study, we also found that the maternal betaine intake alleviated hepatic lipid accumulation, decreased the hepatic levels of TG and TC and increased the mRNA expression of genes involved in lipid oxidation (*PPARα* and *CPT1α*) and transport (*FATP2*) in offspring mice. In addition, we further found the protective effects of maternal betaine on NAFLD and lipid metabolism disorder possibly through reshaping the composition of the gut microbiota and stimulating SCFAs production.

Here, our results showed that maternal betaine supplementation significantly reduced the relative abundance of *Proteobacteria*, *Desulfovibrio*, and *Ruminococcus* in offspring mice, elevated by MHFD. *Proteobacteria* have been proven to be positively related to NAFLD severity, including lobular and portal inflammation, liver fibrosis and the NAFLD Activity Score (NAS) score [34]. *Desulfovibrio*, which belongs to the *Proteobacteria* phylum, can produce lipopolysaccharide, and positively correlated with inflammation and hyperlipidemic [35,36]. Animal experiment also found that the *Desulfovibrio* richness increased along NAFLD–HCC progression [37]. The findings on the reduction in *Desulfovibrio* in 10-week-old offspring mice in our study was consistent with 4-week-old offspring mice with maternal betaine supplementation [23]. In addition, the increment of *Firmicutes* abundance was common in NAFLD patients [38]. The *Ruminococcus* genus belongs to the *Firmicutes* phylum. Boursier et al. found that enrichment of *Ruminococcus* positively correlates with liver fibrosis, and this relationship was independent of metabolic factors [10]. Our correlation study revealed that the abundance of *Proteobacteria*, *Desulfovibrio* and *Ruminococcus* showed a positive relationship with the hepatic TG level, the mRNA expression of genes involved in lipid synthesis (*FAS*) and inflammation (*TNFα*), and a negative relationship with the mRNA expression of genes involved in fatty acid oxidation (*PPARα* and *CPT1α*) and transport (*APOB48* and *FATP2*).

Furthermore, we found that maternal betaine intake increased the relative abundance of *Bacteroides* and *Parabacteroides* in offspring mice, which were reduced by MHFD. Studies have found that the abundance of *Bacteroides* and *Parabacteroides* within the *Bacteroidetes* phylum was relatively lower in patients with obesity and NAFLD [39,40]. The reduction in *Bacteroides* and *Parabacteroides* may indicate an increased risk of NAFLD. The relative abundance of *Bacteroides* decreased with the disease progressing from non-alcoholic fatty liver to non-alcoholic steatohepatitis [40]. Oral administration of live Parabacteroides presented multiple benefits, including improved body weight, glucose homeostasis and hepatic steatosis (macrosteatosis, hepatocyte ballooning and intrahepatic triglyceride), in both ob/ob mice and HFD intervention mice [41]. Consistently, our correlation analysis further found the abundance of *Bacteroides* and *Parabacteroides* was positively related to the mRNA expression of *PPARα* and *CPT1α*, and negatively related to *TNFα* mRNA expression and the hepatic TG level. These above findings in the gut microbiota indicate that a reduction in *Proteobacteria*, *Desulfovibrio*, and *Ruminococcus* and an induction of *Bacteroides* and *Parabacteroides* might be ameliorative mechanisms of maternal betaine on NAFLD in adult offspring.

The altered composition of the gut microbiota might further change the composition of its metabolites, such as SCFAs, TMAO and bile acid. SCFAs are produced by the gut microbiota through fermentation of carbohydrates, which plays an important role in energy metabolism and blood glucose regulation [24]. Lower concentrations of SCFAs (acetic acid, propionic acid, butyric acid and valeric acid) were positively associated with the development of NAFLD [42]. Here, we found that MHFD significantly reduced the levels of total SCFAs, acetic acid, butyric acid and valeric acid in offspring mice, and we further found a positive relationship between the hepatic TG level and the levels of butyric acid and valeric acid. Du et al. demonstrated that betaine supplementation increased the concentrations of acetic acid and butyric acid in the feces of HFD-fed mice [20]. Our study further presented that maternal betaine supplementation increased the levels of acetic acid, butyric acid and valeric acid in the feces of offspring mice exposed to MHFD. The in vitro and in vivo experiments have demonstrated that the SCFAs can play the role of anti-NAFLD through regulation of lipid metabolism. In 6-week-old male C57BL/6J mice, the hepatic levels of TC and TG were significantly decreased, and the mRNA expression of *PPARα* and its downstream enzymes (*ACO* and *CPT1α*) was upregulated by either a low-dose or a high-dose acetic acid intervention for 42 days [43]. The in vitro experiment also proved that acetic acid, propionic acid and butyric acid treatments suppress the accumulation of free fatty acids and TG by decreasing the gene expression of *FAS* and *ACC* and increasing the gene expression of *PPARα* and *CPT1α* [44]. Consistent with these studies, we found that the increment of acetic acid and butyric acid was accompanied by upregulation of the gene expression of hepatic lipid oxidation (*PPARα* and *CPT1α*) and transport (*FATP2*), as well as the downregulation of lipogenic genes (*FAS* and *ACC*). Furthermore, we found a positive relationship between valeric acid and the mRNA expression of *CPT1α* and *FATP2*. The inflammatory response also participates in the development of NAFLD [45]. Previous studies have presented the inverse association between SCFAs and liver inflammation, and supplementation of acetate and butyrate can decrease *TNFα* mRNA expression [46,47]. In our study, correlation analysis also showed that the feces levels of SCFAs (acetic acid, butyric acid and valeric acid) were negatively correlated with hepatic *TNFα* mRNA expression. TMAO and bile acid are the other two critical metabolites in the gut–liver axis [48], while maternal betaine had no significant effect on serum TMAO concentration and bile acid contents in the liver and serum in our study. Thus, SCFAs might be the potential underlying mechanism in the protective effects of maternal betaine on NAFLD in adult offspring.

In summary, our study, for the first time, presented that maternal betaine intake ameliorates MHFD-induced NAFLD by regulating the composition of the gut microbiota and modulating the production of SCFAs in offspring mice. In our study, we offered new insight into the beneficial effects of maternal betaine supplementation in MHFD-related NAFLD in offspring based on the regulation of the gut–liver axis, and reinforced the value of maternal betaine as an efficient intervention for the intergenerational prevention or treatment of NAFLD.

## Figures and Tables

**Table 1:** Sequences of primers used for RT-qPCR.

Gene	Forward Primer	Reverse Primer
*FAS*	GCTGCGGAAACTTCAGGAAAT	AGAGACGTGTCACTCCTGGACTT
*ACC*	CACTGTGAACATGTGGAGG	AGGCTGATGGTGATGACC
*PPARα*	GGGCAAGAGAATCCACGAAG	GTTGTTGCTGGTCTTTCCCG
*CPT1α*	CGCACGGAAGGAAAATGG	TGTGCCCAATATTCCTGG
*APOB48*	GCATGAGTATGCCAATGGTCTCC	CTGGTTGCCATCTGAAGCCATG
*FATP2*	TTCAACAGCGGAGACCTCCTGA	CCACGATGTCAGCGACTTCTGT
*TNFα*	GGTGCCTATGTCTCAGCCTCTT	GCCATAGAACTGATGAGAGGGAG
*IL-1β*	TTCAGGCAGGCAGTATCACTC	GAAGGTCCACGGGAAAGACAC
*ZO-1*	ATCCCACAAGGAGCCATTCC	TAGGGTCACAGTGTGGCAAG
*Occludin*	TTTGCTGTGAAAACCCGAAGA	ACTGTCAACTCTTTCCGCATA
*GPR41*	TTGCTAAACCTGACCATTTCGG	GATAGGCCACGCTCAGAAAAC
*GPR43*	ACAGTGGAGGGGACCAAGAT	GGGGACTCTCTACTCGGTGA
*β-actin*	GGGTCAGAAGGACTCCTATG	GTAACAATGCCATGTTCAAT

**Table 2:** Effects of maternal betaine supplementation on organ coefficients and serum biochemical parameters in offspring mice.

Parameters	CON	HFD	HFD-BET
Liver weight (g)	1.08 ± 0.06	1.31 ± 0.03 *	1.14 ± 0.03 ^#^
Liver/body weight ratio (%)	6.24 ± 0.13	6.64 ± 0.09 *	5.97 ± 0.11 ^###^
Visceral fat weight (g)	0.11 ± 0.02	0.23 ± 0.02 ***	0.17 ± 0.02 ^#^
Visceral fat /body weight ratio (%)	0.59 ± 0.08	1.14 ± 0.07 ***	0.85 ± 0.08 ^#^
ALT (U/L)	29.25 ± 2.63	42.75 ± 4.55 *	33.71 ± 1.21
AST (U/L)	139.25 ± 5.92	210.00 ± 18.20 *	147.43 ± 11.49 ^#^
HDL-C (mmol/L)	1.91 ± 0.21	2.10 ± 0.06	1.79 ± 0.09
LDL-C (mmol/L)	0.36 ± 0.02	0.39 ± 0.02	0.49 ± 0.03
TC (mmol/L)	1.63 ± 0.07	2.55 ± 0.08 **	2.18 ± 0.15
TG (mmol/L)	0.25 ± 0.02	0.34 ± 0.02 *	0.38 ± 0.02
GLU (mmol/L)	8.15 ± 0.96	12.21 ± 2.09	10.30 ± 2.84

Data are presented as the means ± SEM. * *p* < 0.05, ** *p* < 0.01 and *** *p* < 0.001 for the HFD group vs. the CON group; ^#^ *p* < 0.05 and ^###^ *p* < 0.001 for the HFD-BET group vs. the HFD group. Abbreviation: CON group, control diet group; HFD group, high-fat diet group; HFD-BET group, high-fat diet + 1% betaine group; ALT, alanine aminotransferase; AST, aspartate aminotransferase; TG, triglyceride; TC, total cholesterol; HDL-C, high-density lipoprotein cholesterol; LDL-C, low-density lipoprotein cholesterol; GLU, glucose.

## Data Availability

Data supporting reported results are available upon reasonable request and in accordance with the ethical principles.

## Supplementary Materials

The following supporting information can be downloaded at: https://www.mdpi.com/article/10.3390/nu15020284/s1, Figure S1: The consumption of water and betaine in dams; Figure S2: The average litter size of mice. Figure S3: The results of ITT in dams. Figure S4: Effects of betaine intake on gut microbiota in dams. Table S1: The effects of betaine supplementation on organ coefficients and serum biochemical parameters in dams.
