# Supplementary material for: Maternal Betaine Supplementation Mitigates Maternal High Fat Diet-Induced NAFLD in Offspring Mice through Gut Microbiota
Source: Nutrients. 2023 Jan 6;15(2):284. doi: 10.3390/nu15020284 (PMC9861146; doi:10.3390/nu15020284)
Supplement: Supplementary file 1 [file nutrients-15-00284-s001.zip › nutrients-2090207-supplementary.pdf]

Supplemental file

Supporting Information Including (Table S1, Figure S1-S4).

Table S1. The effects of betaine supplementation on organ coefficients and serum biochemical parameters in dams

Parameters	CON	HFD	HFD-BET
Liver weight (g)	1.51±0.07	1.57±0.04	1.40±0.03$^{##}$
Liver/body weight ratio (%)	6.14±0.21	5.74±0.10	5.24±0.10$^{#}$
Visceral fat weight (g)	0.17±0.01	0.44±0.08*	0.34±0.02
Visceral fat /body weight ratio (%)	0.67±0.04	1.72±0.30*	1.28±0.07
ALT (U/L)	28.00±2.08	35.00±5.69	35.00±1.15
AST (U/L)	106.50±10.50	159.60±21.40	111.00±17.24
HDL-C (mmol/L)	1.61±0.19	2.53±0.15*	2.27±0.17
LDL-C (mmol/L)	0.34±0.02	0.36±0.04	0.36±0.01
TC (mmol/L)	2.11±0.24	2.96±0.08*	2.81±0.14
TG (mmol/L)	0.24±0.00	0.39±0.04	0.39±0.07
GLU (mmol/L)	9.21±0.92	11.96±0.97	9.10±1.05

Data are presented as the means ± SEM.* $P < 0.05$ for HFD vs. CON group; $^{#}P < 0.05$ and $^{##}P < 0.01$ for HFD-BET vs. HFD group. Abbreviation: CON group, control diet group; HFD group, high-fat diet group; HFD-BET group, high-fat diet + 1% betaine group; ALT, alanine aminotransferase; AST, aspartate aminotransferase; TG, triglyceride; TC, total cholesterol; HDL-C, high-density lipoprotein cholesterol; LDL-C, low-density lipoprotein cholesterol; GLU, glucose.

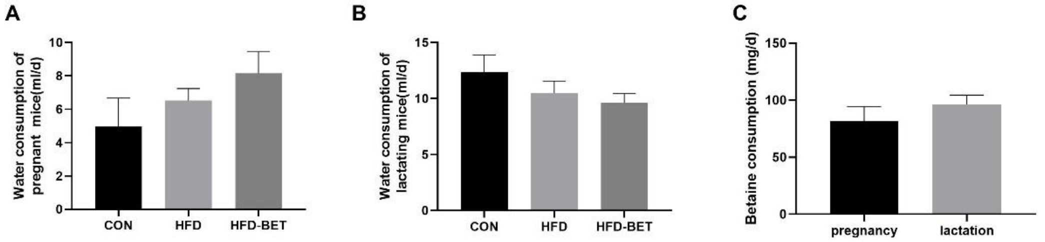

Figure S1. The consumption of water and betaine in dams. (A) Water consumption of pregnant mice; (B) water consumption of lactating mice; (C) betaine consumption of dams during pregnancy and lactation. Data are presented as the means ± SEM. Abbreviation: CON group, control diet group; HFD group, high-fat diet group; HFD-BET group, high-fat diet + 1% betaine group.

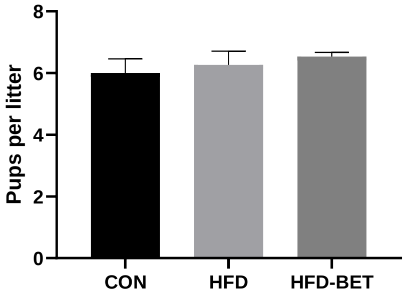

Figure S2. The average litter size of mice. Data are presented as the means ± SEM. Abbreviation: CON group, control diet group; HFD group, high-fat diet group; HFD-BET group, high-fat diet + 1% betaine group.

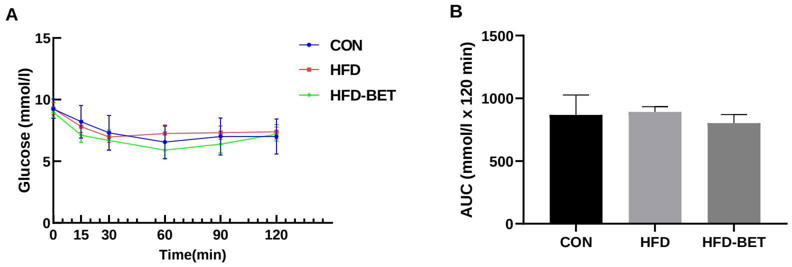

Figure S3. The results of ITT in dams. (A) Serum glucose levels of ITT test; (B) the area under curve (AUC) of ITT test. Data are presented as the means ± SEM.

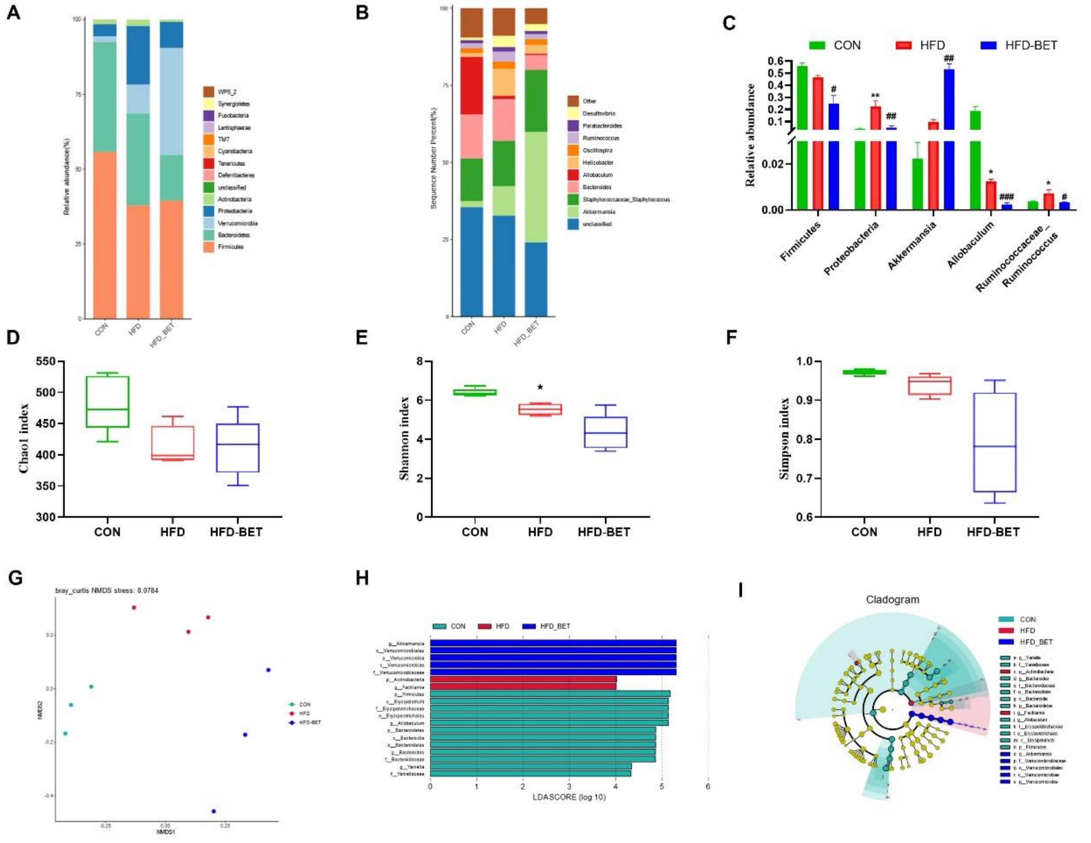

Figure S4. Effects of betaine intake on gut microbiota in dams. (A) Taxonomic composition distribution at the phylum level; (B,C) Taxonomic composition distribution at the genus level; (D-F) Alpha diversity analysis; (G) Non-metric dimensional scaling (NMDS) analysis; (H,I) linear discriminant analysis effect size (LEfSe) analysis, and the linear discriminant analysis (LDA) score using a threshold score larger than 4.0. Data are presented as the means ± SEM. * $P < 0.05$ and ** $P < 0.01$ for HFD vs. CON group; $^{\#}$ $P < 0.05$, $^{\#\#}$ $P < 0.01$ and $^{\#\#\#}$ $P < 0.001$ for HFD-BET vs. HFD group. Abbreviation: CON group, control diet group; HFD group, high-fat diet group; HFD-BET group, high-fat diet + 1% betaine group.
